# Supplementary material for: Improving Dietary Intake of Essential Nutrients Can Ameliorate Inflammation in Patients with Diabetic Foot Ulcers
Source: Nutrients. 2022 Jun 9;14(12):2393. doi: 10.3390/nu14122393 (PMC9228459; doi:10.3390/nu14122393)
Supplement: Supplementary file 1 [file nutrients-14-02393-s001.zip › nutrients-1753185-supplementary.pdf]

Table S1. Nutrient composition of one serving (237 ml) of the nutritional supplement.

Nutrient	Amount
Calories(kcal)	250
calories from fat(kcal)	110
Total Fat(g)	12
saturated Fat(g)	1.5
trans Fat(g)	0
cholesterol(mg)	<5
Sodium(mg)	270
Potassium(mg)	260
Total Carbohydrate(g)	23
dietary Fiber(g)	3
sugars(g)	6
Protein[1](g)	14
Vitamin A[2](IU)	1250
Vitamin C (mg)	102
Calcium (mg)	250
Iron (mg)	3.6
Vitamin D (IU)	240
Vitamin E (IU)	33
Vitamin K (mcg)	16
Thiamin (mg)	0.3
Riboflavin (mg)	0.34
Niacin (mg)	4
Vitamin B6 (mg)	0.4
Folic Acid (mcg)	80
Vitamin B12 (mcg)	1.2
Biotin (mcg)	60
Pantothenic Acid (mg)	2
Phosphorus (mg)	200
Iodine (mcg)	30
Magnesium (mg)	80
Zinc (mg)	3
Selenium (mcg)	14
Copper (mg)	0.4
Manganese (mg)	0.4
Chromium (mcg)	24
Molybdenum (mcg)	15
Chloride (mg)	204
L-carnitine (mg)	25
Taurine (mg)	20
Inositol (mg)	200

[1] Includes protein from caseinate and L-arginine [2] Includes 45% of vitamin A activity from beta-carotene.
